# Evolution of Mn_1−*x*_Ge_*x*_Bi_2_Te_4_ Electronic Structure under Variation of Ge Content

**DOI:** 10.3390/nano13142151

**Published:** 2023-07-24

**Authors:** Tatiana P. Estyunina, Alexander M. Shikin, Dmitry A. Estyunin, Alexander V. Eryzhenkov, Ilya I. Klimovskikh, Kirill A. Bokai, Vladimir A. Golyashov, Konstantin A. Kokh, Oleg E. Tereshchenko, Shiv Kumar, Kenya Shimada, Artem V. Tarasov

**Affiliations:** 1 Department of Physics, Saint Petersburg State University, St. Petersburg 198504, Russia; 2 Donostia International Physics Center, 20018 Donostia-San Sebastián, Spain; 3 Synchrotron Radiation Facility SKIF, Boreskov Institute of Catalysis, Siberian Branch, Russian Academy of Sciences, Kol’tsovo 630559, Russia; 4 Rzhanov Institute of Semiconductor Physics, Siberian Branch, Russian Academy of Sciences, Novosibirsk 630090, Russia; 5 Sobolev Institute of Geology and Mineralogy, Siberian Branch, Russian Academy of Sciences, Novosibirsk 630090, Russia; 6 Hiroshima Synchrotron Radiation Center, Hiroshima University, Hiroshima 739-0046, Japan

**Keywords:** magnetic topological insulator, topological surface states, electronic structure, topological phase transition, topological vertical heterostructure, ARPES, ab-initio calculations

## Abstract

One of the approaches to manipulate MnBi${}_{2}$Te${}_{4}$ properties is the magnetic dilution, which inevitably affects the interplay of magnetism and band topology in the system. In this work, we carried out angle-resolved photoemission spectroscopy (ARPES) measurements and density functional theory (DFT) calculations for analysing changes in the electronic structure of Mn${}_{1-x}$Ge${}_{x}$Bi${}_{2}$Te${}_{4}$ that occur under parameter x variation. We consider two ways of Mn/Ge substitution: (i) bulk doping of the whole system; (ii) surface doping of the first septuple layer. For the case (i), the experimental results reveal a decrease in the value of the bulk band gap, which should be reversed by an increase when the Ge concentration reaches a certain value. Ab-initio calculations show that at Ge concentrations above 50%, there is an absence of the bulk band inversion of the Te $p_{z}$ and Bi $p_{z}$ contributions at the Γ-point with significant spatial redistribution of the states at the band gap edges into the bulk, suggesting topological phase transition in the system. For case (ii) of the vertical heterostructure Mn${}_{1-x}$Ge${}_{x}$Bi${}_{2}$Te${}_{4}$/MnBi${}_{2}$Te${}_{4}$, it was shown that an increase of Ge concentration in the first septuple layer leads to effective modulation of the Dirac gap in the absence of significant topological surface states of spatial redistribution. The results obtained indicate that surface doping compares favorably compared to bulk doping as a method for the Dirac gap value modulation.

## 1. Introduction

The intentional incorporation of impurities into a pristine crystal is a widely employed approach for precisely adjusting the properties of many different systems including topological insulators (TIs) [1,2]. TIs attract considerable attention of researchers due to the phenomenon of band inversion which gives rise to the emergence of topological surface states (TSS) [3] characterized by unique electronic and spin structures [4]. These TSS form a structure referred to as the Dirac cone and exhibit remarkable resistance to scattering induced by non-magnetic impurities [5]. Upon the introduction of magnetism into such a system, a Dirac gap emerges within the TSS [6]. This leads to the possibility of realising unique effects like the quantum anomalous Hall effect [7], which holds promise for applications in quantum computing and spintronics [8]. In intrinsic antiferromagnetic TI, MnBi${}_{2}$Te${}_{4}$, magnetic atoms (Mn) are directly embedded within the crystal structure, thereby enabling a significant internal exchange field that impacts the TSS [9]. MnBi${}_{2}$Te${}_{4}$ is composed of septuple layer (SL) blocks that are separated by van der Waals intervals [10]. Each SL consists of Te-Bi-Te-Mn-Te-Bi-Te atoms. Within the layer, Mn atoms are ferromagnetically coupled, while between adjacent SLs, there is an antiferromagnetic coupling. The development of devices based on quantum anomalous Hall effect necessitates a comprehensive understanding of the factors influencing the energy gap and the capability to modulate it.

A number of works have been devoted to the theoretical investigation of the factors that influence the energy gap [11,12,13]. For instance, a number of works demonstrated that the modulation of the energy gap value is achievable by modifying the surface van der Waals interval in TI crystals [14,15,16]. Furthermore, the modulation of the spin–orbit coupling (SOC) value has proven to be an efficacious approach [11]. The latter is attributed to the fact that the fundamental mechanism driving the emergence of TSS relies on band inversion, which is a direct result of the considerable SOC induced by atoms forming the compound [17]. In view of the fact that the formation of the energy gap in the TSS is driven by the introduction of magnetism into the system, it is crucial to consider the interplay between the effective magnetic field and the Dirac gap value. In this context, the other effective approach for altering the value of the energy gap involves the substitution of transition metal (Mn) atoms with non-magnetic elements. Specifically, elements such as Sn, Pb, and Ge have been investigated as potential substitutes for Mn due to their ability to form the desired ternary compounds, including SnBi${}_{2}$Te${}_{4}$ [18,19], PbBi${}_{2}$Te${}_{4}$ [20], and GeBi${}_{2}$Te${}_{4}$ [21,22], which possess crystal structures similar to MnBi${}_{2}$Te${}_{4}$. Experimental findings have revealed that the replacement of Mn with either Pb [23], Sn [24,25] or Ge [26,27] not only reduces the magnetic moments of the system, but also induces variation in the band structure due to the changes in orbital composition of valence and conduction bands.

This paper aims to analyse the Mn${}_{1-x}$Ge${}_{x}$Bi${}_{2}$Te${}_{4}$ system at various values of the parameter x. Considering that MnBi${}_{2}$Te${}_{4}$ and GeBi${}_{2}$Te${}_{4}$ possess distinct topological invariants ([1;000] and [1;001], respectively [27]), the Mn${}_{1-x}$Ge${}_{x}$Bi${}_{2}$Te${}_{4}$ system is anticipated to undergo a topological phase transition (TPT) at some x value. It ought to be noted that in MnBi${}_{2}$Te${}_{4}$, the band inversion occurs at the Γ-point, while for GeBi${}_{2}$Te${}_{4}$, it takes place at the Z-point [28]. Moreover, a crucial aspect of investigating TIs is the search for new systems that offer appropriate properties for practical applications. This work explores combining MnBi${}_{2}$Te${}_{4}$ and GeBi${}_{2}$Te${}_{4}$ systems to find out new kinds of topological magnetic systems. Such systems could exhibit a diverse range of electronic structure characteristics, making them promising candidates for applications in quantum technologies. As part of this study, we propose and investigate an MnBi${}_{2}$Te${}_{4}$-based system in which the magnetic metal (Mn) atoms in the first surface SL are selectively substituted with non-magnetic elements (Ge). The purpose is to analyse the effects of this controlled local substitution on the electronic structure of the system.

## 2. Materials and Methods

The Mn${}_{1-x}$Ge${}_{x}$Bi${}_{2}$Te${}_{4}$ single crystals under the study were grown using the Bridgman method. These samples are solid solutions of compounds MnBi${}_{2}$Te${}_{4}$ and GeBi${}_{2}$Te${}_{4}$, which possess rhombohedral crystal lattice, with symmetry group $R\bar{3}m$, and are similar to tetradimites (Bi${}_{2}$Te${}_{3}$). Accordingly, the plane of surface (0001) is the plane of easy cleaving for them.

Angle-resolved photoemission spectroscopy (ARPES) measurements were carried out in the Rzhanov Institute Of Semiconductor Physics SB RAS (Novosibirsk, Russia) on the SPECS ProvenX-ARPES facility using He I${}_{\alpha }$ (hν=21.2 eV) radiation for ARPES and hν=1486.7 eV for XPS. Part of the ARPES data were measured on the μ-Laser ARPES system at HiSOR (Hiroshima, Japan) with high spatial resolution (spot diameter of about 5–10 μm) using a Scienta R4000 analyser. In the experiment, the photon energy was hν=6.3 eV, and the maximum photon flux was ∼$10^{14}$ photons/s. The angle between the incident light and the surface normal was 50° [29,30]. The clean surface of the samples was obtained with the cleavage procedure under ultrahigh-vacuum conditions. The base pressure in the chamber was about $5\times 10^{-11}$ Torr.

The electronic structure supercell calculations with impurities were performed using the OpenMX software code (Ver. 3.9.9), which provides a fully relativistic electron density functional theory (DFT) including pseudoatomic orbitals [31,32,33] and norm-conserving pseudopotentials [34]. Calculations were performed in terms of the Perdew–Burke– Ernzerhof generalized-gradient approximation [35]. The surface calculations were performed using 6 SLs slabs of MnBi${}_{2}$Te${}_{4}$ separated by 12 $\overset{{}_{\circ }}{\;A}$ of vacuum. As an unit cell we used the structure presented in the work [16]. For the impurity calculations, 2×2 slab supercells were employed, which allow to consider four non-equivalent positions of Mn atoms within each layer. Ge concentrations of 25, 50, 75 and 100% were obtained by replacing one, two, three and four Mn atoms with Ge atoms, respectively. In all calculations, the AFM state of the systems was considered. The real-space numerical integration grid was determined by a cutoff energy of 450 Ry. The energy convergence criterion was set to be equal to $1\times 10^{-6}$ eV. The k-meshes for Brillouin zones were specified as follows: 5×5×1 mesh for pristine MnBi${}_{2}$Te${}_{4}$ slab and 3×3×1 mesh for 2×2 supercells. The basis functions were set as follows: Bi8.0−s3p2d2f1, Te7.0−s3p2d2f1, Mn6.0−s3p2d1, Ge7.0−s3p2d2 (the pseudopotential cutoff radius is followed by a basis set specification). The Mn 3d states were treated within the DFT+U approach [35] in Dudarev’s scheme with U=5.4 eV [9], which provides a Mn magnetic moment value equal to 5 $\mu_{B}$ and ensures energy positions of the d-states of Mn are far from the Fermi level.

## 3. Results and Discussion

### 3.1. Experimental Results

**Figure 1:**
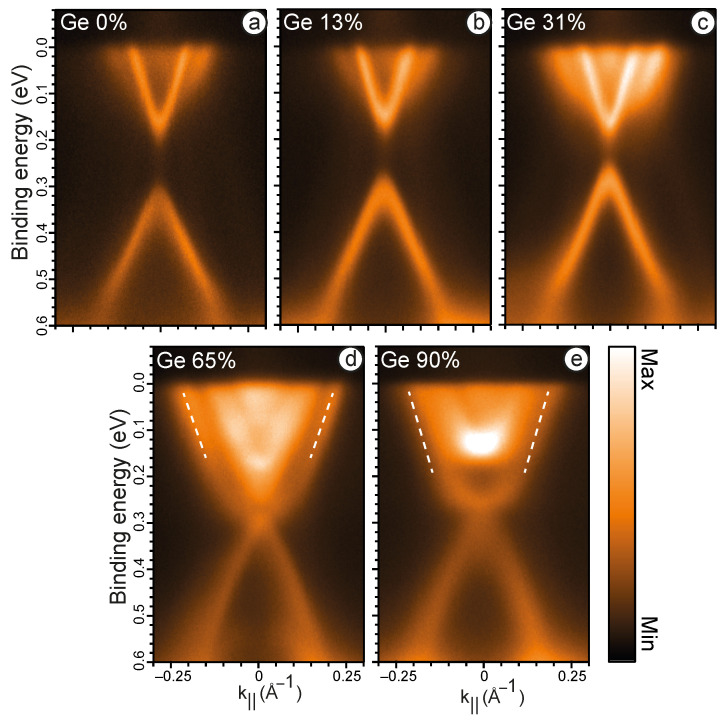
ARPES spectra of Mn${}_{1-x}$Ge${}_{x}$Bi${}_{2}$Te${}_{4}$ crystal with x = 0 (**a**), 0.13 (**b**), 0.31 (**c**), 0.65 (**d**), 0.9 (**e**) measured at T=76 K using hν = 21.2 eV. The white dashed lines depict Ge-derived states.

Firstly, we discuss the bulk doping of the MnBi${}_{2}$Te${}_{4}$ system. Figure 1 shows the ARPES data for the Mn${}_{1-x}$Ge${}_{x}$Bi${}_{2}$Te${}_{4}$ system with x = 0, 0.13, 0.31, 0.65, 0.9. These spectra provide valuable insights into the modification of the electronic structure of Mn${}_{1-x}$Ge${}_{x}$Bi${}_{2}$Te${}_{4}$ induced by Mn substitution with Ge. The Ge concentrations were derived from XPS data. The ARPES data for all x values were obtained using an excitation energy of 21.2 eV (HeIα emission line). It is important to highlight that at this excitation energy, cross section for the TSS of MnBi${}_{2}$Te${}_{4}$ is rather low. In such measurements, the bulk conduction and valence states are predominantly visible. Figure 1a depicts the band dispersion nearby the Γ-point of the intrinsic TI MnBi${}_{2}$Te${}_{4}$, i.e., the case of Ge 0%. The data presented are consistent with previously reported measurements of MnBi${}_{2}$Te${}_{4}$ [9], demonstrating a bulk band gap of approximately 180 meV. These findings align with earlier studies [36] and confirm the reproducibility of the obtained results. The results presented in Figure 1b illustrate the electronic structure of Mn${}_{0.87}$Ge${}_{0.13}$Bi${}_{2}$Te${}_{4}$. Because of the finite width of the PE spectral characteristics, it is challenging to assess whether the gap slightly shrinks or not. Upon further increasing the concentration of Ge to 31% (Figure 1c), the bulk band gap exhibits a gradual reduction, eventually reaching a value of about 140 meV. This can be attributed to the modulation of the effective SOC strength induced by changes in the Bi-p contributions in the bulk gap edges, as it was suggested in Ref. [27]. Once Ge concentration reaches 65% (Figure 1d), a closure of the energy gap ensues, concomitant with an intensified prominence of the states, modified by Ge contribution to the system–Ge-derived states (indicated by white dashed lines). With further increases in concentration up to 90% (Figure 1e) these states acquire the form extremely similar to TSS of GeBi${}_{2}$Te${}_{4}$ [21,22], localised within the bulk band gap in the energy range from 0.15 to 0.3 eV. Hence, at a certain x value there is a reopening of the bulk band gap.

Thus, the experimental results obtained in this study reveal a decrease in the value of the bulk band gap (up to a Ge concentration of 65%), which should be reversed by an increase when the Ge concentration is equal or less than 90%. As noted above, MnBi${}_{2}$Te${}_{4}$ and GeBi${}_{2}$Te${}_{4}$ are materials with different topological invariants, so the transition from a decreasing gap to an increasing gap can be related to the possibility of a TPT in the system.

**Figure 2:**
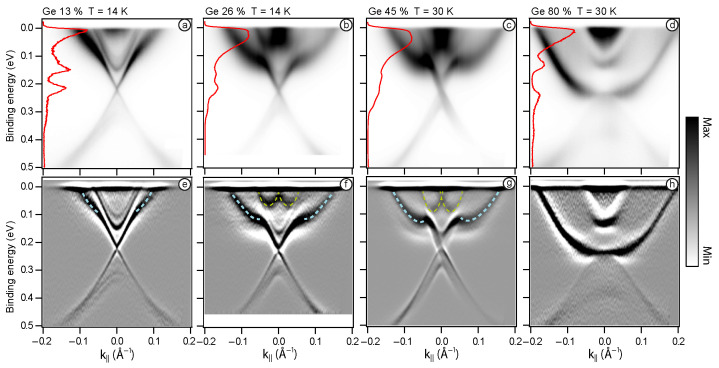
ARPES spectra of Mn${}_{1-x}$Ge${}_{x}$Bi${}_{2}$Te${}_{4}$ crystal for x = 0.13 (**a**,**e**), 0.26 (**b**,**f**), 0.45 (**c**,**g**), 0.8 (**d**,**h**), presented as N(E)-top line and $d^{2}N\left(E\right)/dE^{2}$-bottom line. The blue dashed lines depict Ge-derived states. The yellow dashed lines indicate Rashba-like states. Energy distribution curves at the Γ-point are shown on the left side of the top panels. hν = 6.3 eV.

To analyse the TSS, Figure 2 presents the ARPES data obtained for electron photoexcitation using laser irradiation (hν=6.3 eV) for Mn${}_{1-x}$Ge${}_{x}$Bi${}_{2}$Te${}_{4}$, where x = 0.13, 0.26, 0.45, 0.8. Figure 2a exhibits the band dispersions attributed to the Mn${}_{0.87}$Ge${}_{0.13}$Bi${}_{2}$Te${}_{4}$ system. The observed dispersion of electronic states slightly differs from the electronic structure of MnBi${}_{2}$Te${}_{4}$ [37]. To provide a more detailed characterization of the spectrum, Figure 2e highlights the second derivative $d^{2}N/dE^{2}$, where the presence of the Dirac cone, as well as the valence and conduction band states, is clearly discernible. Due to the finite width of the TSS, it is difficult to judge about the Dirac gap value, but it is apparently less than 70 meV, observed in clear MnBi${}_{2}$Te${}_{4}$ [9]. It should be noted that the increase of Ge concentration does not result in a significant non-magnetic Dirac gap, as for example in the case of Sb-doped MnBi${}_{2}$Te${}_{4}$ [38]. Thus, Ge does not introduce additional defects into the structure leading to electron scattering of TSS. With an increase in Ge concentration up to 26% (Figure 2b,f), the states of the upper part of the Dirac cone undergo broadening, accompanied by distinctive features attributed to the presence of Ge—the Ge-derived states (depicted in blue dashed lines). The experimentally obtained results for the Mn${}_{0.55}$Ge${}_{0.45}$Bi${}_{2}$Te${}_{4}$ are shown the crossing of the upper and lower parts of the cone at the Γ-point, resulting in the formation of a gapless state (Figure 2c,g). Finally, Figure 2d,h represents the electronic structure for the high Ge content (80%). By comparing our results with theoretical studies [18] and experimental investigations [22] of the GeBi${}_{2}$Te${}_{4}$ system, we can state that the ARPES data for the sample with a high Ge concentration exhibit a TSS pattern that is characteristic of the previously measured GeBi${}_{2}$Te${}_{4}$. It is worth mentioning that the introduction of Ge also leads to the Rashba states (displayed as yellow dashed lines) become apparent in the energy range from 0 to 0.1 eV (Figure 2f). It is of importance that these states are observed in pure MnBi${}_{2}$Te${}_{4}$ and are not attributed to the incorporation of Ge into the system. It is assumed that these states are likely quasi-two-dimensional bulk states or surface resonances [39,40]. In the case of 45% Ge content, the Rashba-like states exhibit enhanced visibility, demonstrating a noticeable shift towards higher binding energies. The changes of the electronic structure that occur with increasing Ge content will be analysed further by means of ab-initio calculations.

### 3.2. Theoretical Calculations

#### 3.2.1. Bulk Mn/Ge Substitution

**Figure 3:**
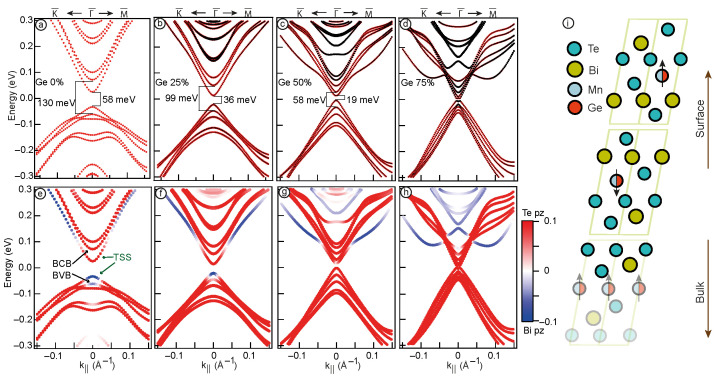
Calculated band structure of the TSS and the nearest valence and conduction band (BVB and BCB) states. The black markers depict Ge contribution (upper panel); the difference between Te $p_{z}$ (red) and Bi $p_{z}$ (blue) contributions (lower panel) for Mn${}_{1-x}$Ge${}_{x}$Bi${}_{2}$Te${}_{4}$ system with x = 0% (**a**,**e**), 25% (**b**,**f**), 50% (**c**,**g**), 75% (**d**,**h**). Structure of the MnBi${}_{2}$Te${}_{4}$ SL with the arrangement of atoms (**i**). The black arrows represent the direction of the magnetic moment.

To investigate in detail the influence of Ge content on the electronic structure of the Mn${}_{1-x}$Ge${}_{x}$Bi${}_{2}$Te${}_{4}$ system, we have performed DFT calculations for MnBi${}_{2}$Te${}_{4}$ TI slab surface model with Mn replaced by Ge. The calculations were executed using a 2×2 supercell, wherein the parameter x was assigned to discrete values of x = 0, 0.25, 0.5, and 0.75. The substitution of Mn by Ge was made in all Mn layers of the slab. It was found that although different variants of the placement of Ge atoms in Mn layers can in some way change the general view of the electronic structure, nevertheless, the electronic states near the Fermi level in various variants remain a very similar. The results are collected in Figure 3, where panels in upper row show the Ge contribution in electron states depicted in black, while the bottom panel illustrates the difference between the Te $p_{z}$ and Bi $p_{z}$ contributions.

Figure 3a displays the Dirac cone as well as the nearest states in the valence and conduction bands for pure MnBi${}_{2}$Te${}_{4}$. The obtained results show a bulk band gap of approximately 130 meV and the Dirac gap of 58 meV, which are consistent with previous calculations for this type of TI [11]. In Figure 3b, the band structure for MnBi${}_{2}$Te${}_{4}$ with 25% Mn substitution by Ge is presented. The obtained results reveal a bulk band gap of 99 meV and a Dirac gap of 36 meV. Similar to the experimental dispersions depicted in Figure 1, the calculated results also demonstrate a reduction in the bulk band gap and indicate a decrease in the Dirac gap. With a further increase of Ge concentration up to 50% (Figure 3c), the bulk band gap continues to decrease, reaching a value of 58 meV. Besides, in the energy range of 0.1–0.3 eV the states corresponding to the Ge bands are observed, and the value of the Dirac gap in this case is 19 meV. It is worth noting that changes in the band structure are accompanied by an increase in the contribution of Ge to electron states. The calculated electronic structure for the Mn${}_{0.25}$Ge${}_{0.75}$Bi${}_{2}$Te${}_{4}$ system, presented in Figure 3d reveals a complex band structure indicating the intersection of the upper and lower parts of the cone at the Γ-point with the formation of avoided crossings. This case is similar to the pattern of the electronic structure observed in the ARPES data for Mn${}_{0.55}$Ge${}_{0.45}$Bi${}_{2}$Te${}_{4}$ (Figure 2c). The Ge contribution, represented by the highlighted black color, becomes even more prominent, and the manifestation of Rashba-like states takes place.

Note that in our DFT calculations, we neglected such factors as structural changes occurring when varying Ge content in the Mn${}_{1-x}$Ge${}_{x}$Bi${}_{2}$Te${}_{4}$ system and the inevitable presence of antisite defects in such systems [15,41]. In addition, the disadvantage of the slab approach is the presence of finite-size effects and artificial ordering of impurities in the structure [42,43,44]. In this regard, we tend to believe that our calculation results cannot be directly compared with experimental results for the same concentrations. Nevertheless, our results allow us to draw convincing conclusions about the influence of the single factor—an increase in Ge concentration on the electronic structure of Mn${}_{1-x}$Ge${}_{x}$Bi${}_{2}$Te${}_{4}$. Therefore, one can state that the incorporation of Ge into the Mn${}_{1-x}$Ge${}_{x}$Bi${}_{2}$Te${}_{4}$ TI system has a significant impact on its electronic structure. This influence is that both the Dirac gap and the bulk band gap are being reduced as Ge content increases. This fact cannot be explained only by the decrease in the magnetic moment of the system resulting from the substitution of Mn with Ge. Since the value of the bulk band gap is determined by the SOC strength, the orbital contribution of Ge should also plays a significant role, as evidenced by the Ge-derived states observed in Figure 3b–d. Moreover, the Dirac gap can be influenced by the spatial distribution of TSS, which can changes as the Ge concentration increases, and this factor will be subject to detailed analysis in the subsequent discussion. Importantly, both experimental and theoretical investigations consistently demonstrate a reduction in the bulk band gap as the Ge concentration increases. Hence, the observed changes in the slab calculation results are consistent with the experimental data, indicating a certain agreement between theory and experiment.

The criteria for the presence of a topological state is the inversion of the valence and conduction bands [1]. For MnBi${}_{2}$Te${}_{4}$, it appears as a domination of the contribution of the Bi $p_{z}$ states at the top edge of the valence band, and of the Te $p_{z}$ states at the bottom edge of the conduction band [19,23]. Figure 3e–h shows the calculated dispersion dependencies for different concentrations of Ge, in which the colour shows the difference in contributions of the Te $p_{z}$ states and Bi $p_{z}$ states (red and blue, respectively). Figure 3e depicts the band inversion taking place at the edges of the bulk band gap (marked with black arrows). Besides, there is also a similar orbital configuration of the TSS (marked with green arrows). The predominance of Bi $p_{z}$ contribution within the TSS in the conduction band at high values of k may be related to the presence of the Rashba states as shown in Ref. [45]. Such states can split off from states in the conduction band and strongly hybridise with TSS. As the concentration of Ge increases up to 25% (Figure 3f), there is a decrease in the Bi $p_{z}$ contribution to the edge of bulk valence band which results in the fact that Te $p_{z}$ and Bi $p_{z}$ contributions become nearly equal, indicating the proximity of the system to the point of TPT. When the Ge concentration is further increased to 50% (Figure 3g), the band inversion disappears. This may indicate a TPT that occurs at Ge concentrations between 25 and 50%. At the Ge concentration of 50%, both the bulk states and the TSS demonstrate Te $p_{z}$ domination, so it is difficult to identify the phase of the system. A similar situation is observed when 75% of Mn is substituted with Ge (Figure 3h). In fact, within this concentration range, the possibility of a semimetallic state has been demonstrated in previous studies [27]. Next, we will consider in detail changes in the localisation of the states at the band gap edges.

**Figure 4:**
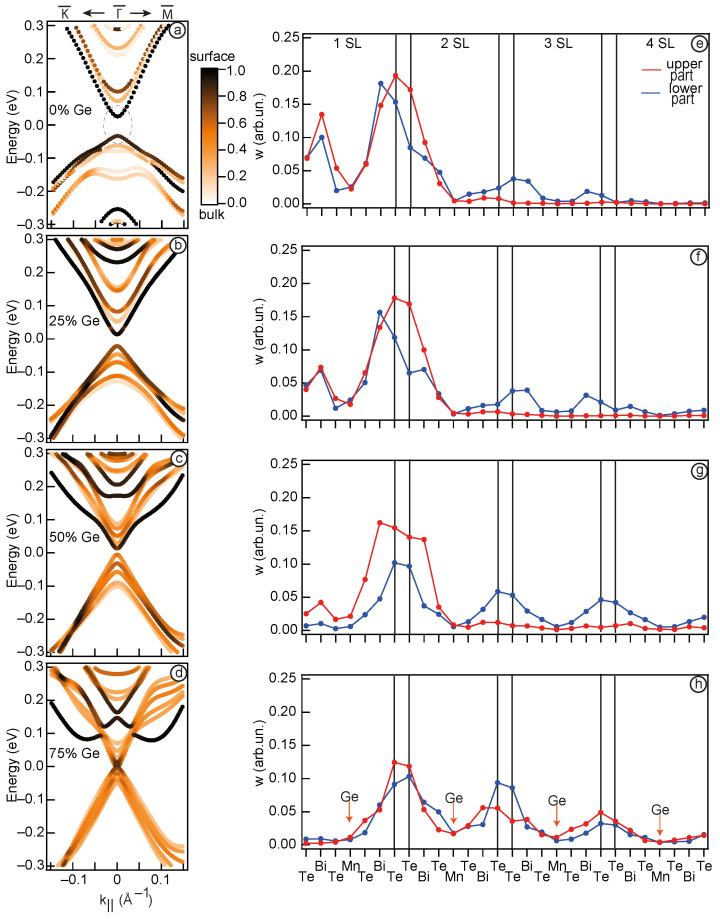
The band structure of Mn${}_{1-x}$Ge${}_{x}$Bi${}_{2}$Te${}_{4}$ system for x = 0% (**a**), 25% (**b**), 50% (**c**), 75% (**d**). The color scale indicate the surface localisation. Distribution in the TSS localisation for the lower and upper parts of the cone (blue and red points, respectively) (**e**–**h**). In panel (**a**), the dotted circle indicates the area for which localisation was calculated. For all x values the k range from −0.01 to 0.01 ${Å}^{-1}$ was used.

The left column of Figure 4 provides the electronic structure of the calculated systems with the marked surface contribution (highlighted for the first two surface SL). The right column of Figure 4 depicts a visual representation of the upper and lower parts of the cone-shape states spatial distribution, denoted by the red and blue colors, respectively, for a system where Mn/Ge substitution is made throughout the entire slab, consisting of 6 SLs. Figure 4a displays the case of pure MnBi${}_{2}$Te${}_{4}$. It is shown that the states forming the Dirac cone consist of dark markers, indicating that they are localised at the surface (mostly within 1 SL). When 25% Ge is added to the system, this picture is generally maintained (Figure 4b). When the Ge concentration is further increased to 50% (Figure 4c), the upper part of the cone localised on the surface atoms, while the lower cone is formed prevalently by bulk atoms. Such observations confirm that TSS no longer exist in this case and the system cannot be considered as TI. Finally, at 75% Ge (Figure 4d) the surface contribution to the states at the band gap edges becomes minimal.

For a more detailed analysis of spatial redistribution of these states, we present the localisation as a one-dimensional curve, where the contribution in these states is matched to the atomic order number in the slab. A more detailed description of the method can be found in Ref. [11]. Applying this estimation to the pure MnBi${}_{2}$Te${}_{4}$ case (Figure 4e), we see that the TSS primarily localise at the interface between the first and second SLs, confirming previous findings [11,13]. Moving to Figure 4f, which corresponds to the case of 25% Mn/Ge replacement, it becomes apparent that the gap between VB and CB is formed by surface states, as their localisation is predominantly concentrated within the first 2 SLs. However, as the system reaches 50% Ge concentration (Figure 4c), the states comprising the lower part of the cone-shaped states are no longer localised at the surface, but become distributed throughout the crystal. Notably, the upper part of the cone-shaped states, i.e., Ge-derived states, retains its surface localisation. This change in localisation is accompanied by an absence of the band inversion signs, as depicted in Figure 3g, indicating a transition of the system to a different phase. At 75% Ge concentration (Figure 4d,h), both the upper and lower cone-shape states are distributed across the entire crystal, while Ge-derived states transforms into Rashba-like states.

Thus, in the TI phase (Figure 4a,b), the TSS are primarily localised in the first 2 SLs region, which is characteristic of such states. Observed changes in the spatial localisation of the states at the band gap edges confirm the occurrence of a TPT between 25% and 50% Ge concentration. This is evident in the transition from TSS localised at the surface (in the TI phase) to the states expanding throughout the entire slab.

#### 3.2.2. Surface Mn/Ge Substitution

**Figure 5:**
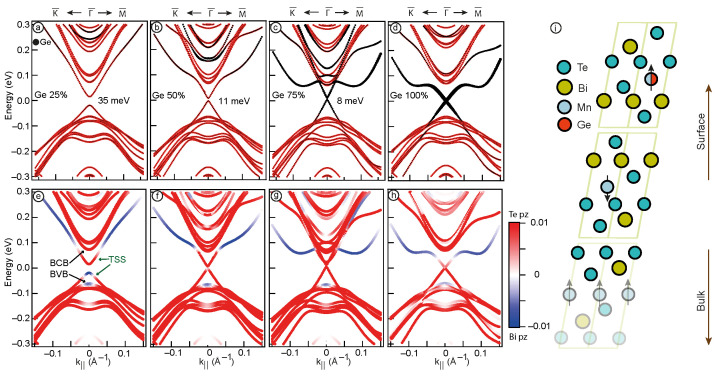
Calculated band structure of the TSS and the nearest valence and conduction band (BVB and BCB) states. The black markers depict Ge contribution (upper panel); the difference between Te $p_{z}$ (red) and Bi $p_{z}$ (blue) contributions (lower panel) for Mn${}_{1-x}$Ge${}_{x}$Bi${}_{2}$Te${}_{4}$/MnBi${}_{2}$Te${}_{4}$ system with x = 0% (**a**,**e**), 25% (**b**,**f**), 50% (**c**,**g**), 75% (**d**,**h**). Structure of the MnBi${}_{2}$Te${}_{4}$ SL with the arrangement of atoms (**i**). The black arrows represent the direction of the magnetic moment.

Since Mn/Ge substitution in the entire slab can lead to a transition from TI to another phase, prospects for the practical applications of such a system remain rather vague. Hence, it is crucial to continue exploring alternative approaches to modulate the properties of MnBi${}_{2}$Te${}_{4}$. A promising method of changing the electronic structure at the Dirac point is the so-called “magnetic extension”, when a film of stoichiometric magnetic TI is created on the surface of a nonmagnetic TI [46,47]. Besides, in order to create new layered topological systems, it is possible to selectively dope only the first SL instead of the entire system [48,49]. To explore the realisation of such layered systems based on magnetic TIs, we investigated the Mn${}_{1-x}$Ge${}_{x}$Bi${}_{2}$Te${}_{4}$/MnBi${}_{2}$Te${}_{4}$ heterostructure with the substitution of Mn by Ge in the first SL. The obtained results are presented in Figure 5.

The theoretical calculations were performed for x values of 0.25, 0.5, 0.75 and 1 (Figure 5a–d, respectively). Figure 5a represents the case of Mn${}_{0.75}$Ge${}_{0.25}$Bi${}_{2}$Te${}_{4}$, where the Dirac gap decreases from 58 meV to 35 meV comparing with pure MnBi${}_{2}$Te${}_{4}$. This reduction can be primary attributed to a decrease in magnetic moment value in the area where TSS are localised. Next, in the Mn${}_{0.5}$Ge${}_{0.5}$Bi${}_{2}$Te${}_{4}$ system, the bulk band gap value remains unchanged, as expected, while the Dirac gap demonstrates a more significant decrease, reaching 11 meV (Figure 5b). Subsequently, as the Mn/Ge substitution increases up to 75%, the Dirac gap further reduces to 8 meV (Figure 5c) and closes when Ge content reaches 100% (Figure 5d). In this case the system corresponds to the interface of GeBi${}_{2}$Te${}_{4}$ thin film on the MnBi${}_{2}$Te${}_{4}$ crystal surface. In the upper panel of Figure 5 the Ge contribution is highlighted in black and is particularly visible in the system with the maximum Ge concentration, i.e., GeBi${}_{2}$Te${}_{4}$/MnBi${}_{2}$Te${}_{4}$ structure (Figure 5d). The results suggest that the modulation of the Dirac gap value is more effective in the system where the substitution of Mn/Ge is confined to the first SL, in comparison to the case where the substitution is carried out throughout the entire system. The differences can be attributed to the fact that, in a heterostructure like this, the material retains its bulk magnetic TI properties at all concentrations of Ge, while its surface SL undergoes modification. This indicates that the TSS in this system persist at all Ge concentrations, although they are influenced by the increasing Ge content in the system. In this way, it is possible to precisely tuning the properties of TSS (by changing the value of the Dirac gap) without changing the bulk electronic structure.

The bottom panel (Figure 5e–h) illustrates the band dispersions with the contributions of Te $p_{z}$ and Bi $p_{z}$ orbitals emphasized. It is noteworthy that as the Ge concentration is progressively increased from 25% to 100%, the inversion of Te $p_{z}$ and Bi $p_{z}$ contributions at the edges of the bulk gap is maintained. This preservation of the inversion indicates that the system retains in non-trivial state. It is worth to note that beyond a Ge concentration of 25%, the dominance of Bi $p_{z}$ in the lower region of the Dirac cone is supplanted by the prevalence of Te $p_{z}$, resembling the analogous behavior observed when doping the entire crystal. We will explore in detail changes in the localisation of the states at the band gap edges in the surface doping case and compare it with the bulk one.

**Figure 6:**
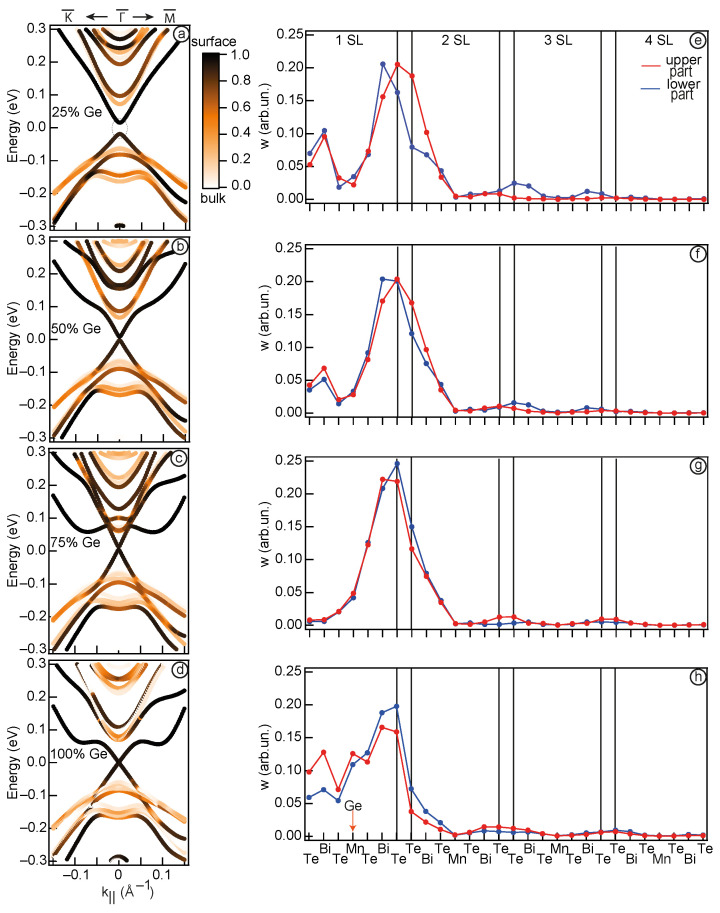
The band structure of Mn${}_{1-x}$Ge${}_{x}$Bi${}_{2}$Te${}_{4}$/MnBi${}_{2}$Te${}_{4}$ system for x = 25% (**a**), 50% (**b**), 75% (**c**), 100% (**d**). The color scale indicates the surface localisation. Distribution in the TSS localisation for the lower and upper parts of the Dirac cone (blue and red points, respectively) (**e**–**h**). In panel (**a**), the dotted circle indicates the area for which localisation was calculated. For all x values the k range from −0.01 to 0.01 ${Å}^{-1}$ was used.

The left column of Figure 6 corresponds to the Mn${}_{1-x}$Ge${}_{x}$Bi${}_{2}$Te${}_{4}$/MnBi${}_{2}$Te${}_{4}$ band structure with the marked surface contribution in the first and second SLs. We can see that at all concentrations of Ge between 25 and 100% (Figure 6a–d), the states at the band gap edges remain their surface localisation. A more detailed analysis of spatial redistribution in this system shows a different (in contrast to the bulk doping) pattern of changes in TSS spatial distribution (Figure 6e–h). It is noteworthy that increasing the Ge concentration up to 75% primarily affects the localisation of TSS at the first two surface atoms (Te and Bi in the first SL) and only the GeBi${}_{2}$Te${}_{4}$/MnBi${}_{2}$Te${}_{4}$ case demonstrate more drastic changes. In general, the observed increase in the TSS localisation at the Ge, at least in the case of the heterostructure, may indicate a some influence of the Ge orbital composition on the TSS electronic structure. Thus, the analysis of the data presented in Figure 6 reveals that replacing Mn/Ge only in the first SL does not result in the redistribution of TSS deep into the crystal. Instead, they maintain their localisation in the first two SL. This is very essential: at any concentration x, the material under study is still a TI, which makes it attractive for device applications. We suppose that the main factor modulating the value of the Dirac gap in this case is the reduction in effective magnetism of the first SL, since there is no significant redistribution of TSS between SLs when the Ge content is increased. Hence, when Ge completely replaces Mn in the first SL, the TSS is no longer affected by magnetism and become gapless. The influence from the underlying SLs is negligible, due to the shift of the TSS localisation into the first SL. The results obtained indicate that surface doping compares favorably with bulk doping in terms of the method of varying the TSS gap value, since material in this case unambiguously retains its topological nature.

## 4. Conclusions

In this paper, we give further insight into the investigation of the magnetic dilution effect in the intrinsic topological insulator MnBi${}_{2}$Te${}_{4}$. The influence of a gradual increase in Ge content in Mn${}_{1-x}$Ge${}_{x}$Bi${}_{2}$Te${}_{4}$ has been studied by ARPES and DFT methods. Both revealed the modification of the surface electronic structure manifested by a decrease in the bulk band gap at the Dirac point. Its monotonic decrease is observed for concentrations up to 50–60%, according to experimental studies. At higher Ge concentrations, the electronic structure of the surface becomes close to the electronic structure of GeBi${}_{2}$Te${}_{4}$. Based on DFT calculations, the possibility of a TPT was shown to occur at Ge concentrations between 25 and 50%. As an indication of the TPT, we consider changes in the orbital composition of the edges of bulk band gap (i.e., inversion of the Te $p_{z}$ and Bi $p_{z}$ states), as well as changes in the localisation of states that at 0% Ge are TSS. It also follows from the calculations, that at a Ge concentration of 75%, the compound is also not a TI. Although our calculation results cannot be directly compared with experimental results for the same concentrations due to their unavoidable constraints, they allow one to draw convincing conclusions about the influence of an increase in Ge on the electronic structure of Mn${}_{1-x}$Ge${}_{x}$Bi${}_{2}$Te${}_{4}$. However, the future prospects of the practical application of this system in devices remain rather vague. In this connection, by means of DFT calculations, we have considered a topological vertical heterostructure consisting of thin film of Mn${}_{1-x}$Ge${}_{x}$Bi${}_{2}$Te${}_{4}$ on MnBi${}_{2}$Te${}_{4}$ crystal substrate. It was demonstrated that the substitution of Mn with Ge in the first SL can effectively modulate the value of the Dirac gap. In contrast to bulk doping, this process should not be accompanied by TPT of the system, and the TSS are conserved at all concentrations of Ge in the system. Therefore, the system with surface doping retains its importance in the development of devices based on magnetic TI.

## Data Availability

Data will be made available on request.
